# Genomic and transcriptomic analyses provide new insights into the complex impacts of bud mutation in peach

**DOI:** 10.1038/s41598-025-07225-w

**Published:** 2025-07-02

**Authors:** Guo Rui, Zhou Ping, Yan Shaobin, Jin Guang, Li Yong

**Affiliations:** 1 Fruit Research Institute, Fujian Academy of Agricultural Sciences, Fuzhou, 350013 China; 2 Zhengzhou Fruit Research Institute, Chinese Academy of Agricultural Sciences, Zhengzhou, 450009 China; 3 Zhongyuan Research Center, Chinese Academy of Agricultural Sciences, Xinxiang, 453000 China

**Keywords:** *Prunus persica*, Bud mutation, Hairless, Genomic variations, RNA-seq, Genetics, Functional genomics

## Abstract

Bud mutation is an important solution in fruit breeding, but the mechanism of bud mutation remains largely unknown. Here, we present the phenotypic, genomic, and transcriptomic analyses of a peach hairless bud mutation. Phenotypic analyses found an increase in the soluble solid content (SSC), fructose, and glucose, as well as a decrease in fruit weight, sucrose, and sorbitol in hairless bud mutation. Genomic analyses showed a higher number of SNPs, indels, SVs, and TEs in the hairless bud mutation than the wild type. Moreover, most of the variations were heterozygous. An LTR transpose element in the third exon of *PpMYB25* was identified in the hairless mutation, but was absent in the wild type, underlying the hairless phenotype. A total of 660 differentially expressed genes were identified in mutation, including 254 were upregulated and 406 were downregulated. The functional annotation of differentially expressed genes revealed two sucrose synthase gene *PpSUS3* and *PpSUS4*, as well as two nitrate transporter gene *PpNRT2.5* and *PpNRT1*, implying the candidates underlying the increase in the SSC and decrease in the fruit weight in mutation. Our results elucidated the complicated impacts of bud mutation on the genome and transcriptome provide new insights into, providing new insights into the molecular mechanism of somatic mutation in plants.

**Supplementary Information:**

The online version contains supplementary material available at 10.1038/s41598-025-07225-w.

## Introduction

Somatic variation is one of the most important ways to generate new variations and broaden genetic diversity^1^. Somatic variations occur in nearly all plant and animal species, especially in fruit tree crops. For the perennial woody fruit trees, somatic variations could lead to a broad spectrum of phenotypes and have been considered to be an important breeding solution^2,3^. For instance, more than 1,300 somatic mutants in sweet orange have been recorded, and some of these mutants have been widely cultivated around the world^4^. However, the molecular basis of how somatic mutations influence gene expression and agronomic traits remains largely unknown.

Peach is one of the most important temperate fruit species, with a production of 25 million tons and a net value of USD 21.6 billion in 2021^5^. Peach is considered to be the model plant for the research of fruit species in the Rosaceae family because of its small genome size, self-compatibility, and short juvenile phase. Several previous studies have identified the genes or loci that confer the agronomic traits, such as the fruit shape, flesh color, flesh texture, chilling requirements, etc^6–10^. Bud mutations in fruit species have been considered as one type of somatic, and have been widely used as an important breeding solution for peach. Many bud mutations, such as the fruit shape and fruit appearance, have been found. For instance, an early-ripening bud mutation cultivar named ‘Li Xia Hong’, from the nectarine cultivar ‘Zhong You 4’, has been released in China^11^.

To reveal the impacts of bud mutation, we performed a comprehensive analysis of the phenotypes, genomes, and transcriptomes of the hairless mutation. We found the significant changes in the fruit size and sugar content in the hairless mutation. The variation map revealed a higher number of variations in the hairless mutation than in the wild type, and most of them were heterozygous. Finally, the candidate genes underlying the changes in the phenotypes were identified.

## Materials and methods

### Plant materials

**Fig. 1:**
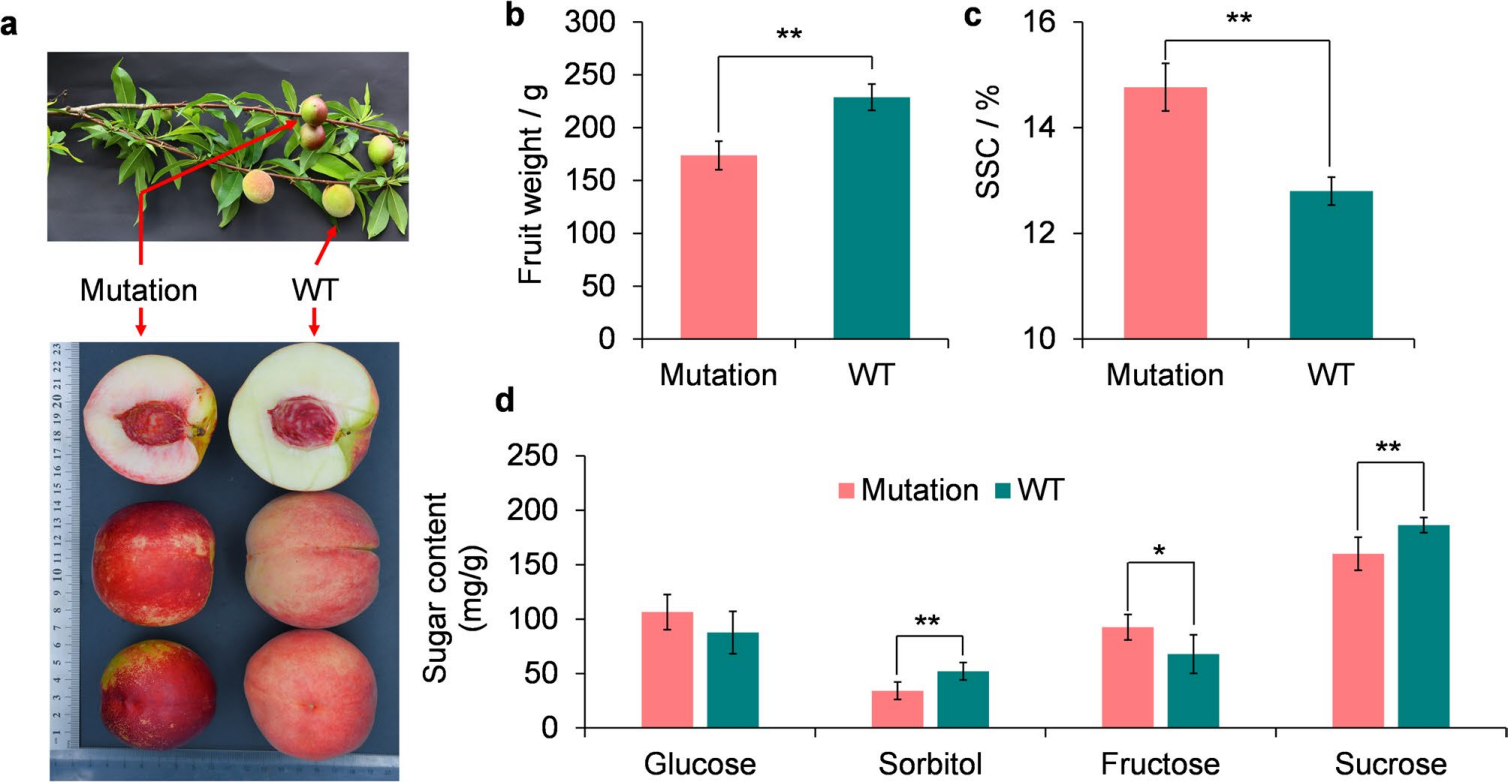
Phenotype changes in hairless bud mutation. (**a**) Image of bud mutation and wild type. (**b**) Comparison of fruit weight between wild type and bud mutation. (**c**) Comparison of SSC between wild type and bud mutation. (**d**) Comparison of glucose, sorbitol, fructose, and sucrose between wild type and bud mutation. * represents *p* < 0.05. ** represents *p* < 0.01.

The five-year-old wild type ‘Zhong Tao 5’ (WT) (Patent No: Yu S-SV-PP-012-2015, patentee: Zhengzhou Fruit Research Institute, Chinese Academy of Agricultural Sciences) and its hairless mutation accessions were planted at the Fruit Research Institute, Fujian Academy of Agricultural Sciences (Fuzhou, Fujian, China). The formal identification of the ‘Zhong Tao 5’ and its mutation were performed by Guo Rui and Jin Guang (Fig. 1a). This material has not been deposited in a publicly available herbarium. RNA-seq samples for the wild type and hairless mutation accessions were collected when they ripened on July 5, with three biological replicates. For each replicate, approximately 5 g of fruit flesh was collected. For genome sequencing, approximately 5 g of young leaves from the wild type and hairless mutation accessions was collected.

### Phenotype investigations

The wild type and bud mutation were cloned with 15 trees by grafting, respectively. Three biological replicates were established for phenotyping. For each biological replicate, a total of five fruits were collected from grafted tree and used to detect the fruit weight and SSC. Fruit sugar extractions were conducted according to a previous study^12^. The sugar type and content detection were performed using high-performance liquid chromatography (HPLC) following a previously reported protocol^13^.

### Sequencing

For genome sequencing, genomic DNA was extracted from young leaves using the cetyltrimethylammonium bromide (CTAB) method, and at least 4 µg of genomic DNA from each accession was used to construct a sequencing library, following the manufacturer’s instructions (Illumina Inc.). Paired-end sequencing libraries with an insert size of approximately 300 bp were sequenced using an Illumina NovaSeq 6000 sequencer, with read lengths of 150 bp. For RNA-seq, the total RNA was extracted using a quick extraction kit (Aidlab, Beijing, China). The double-strand cDNA was synthesized and purified, and the adapters were ligated to the short fragments following the manufacturer’s protocol. The constructed RNA-Seq libraries were sequenced using the Illumina NovaSeq platform in paired-end 150-bp mode.

### Variation calling

The genome sequencing data was aligned to the peach ‘Lovell’ reference genome using BWA (version 0.7.12)^14^with the following parameters: bwa mem -t 4 -M -R. The mapped reads in SAM format were converted into BAM format, sorted according to the mapping coordinates, and processed for PCR duplicate removal using the Picard package (http://broadinstitute.github.io/picard/, version 1.136), with default parameters. SNP and indel callings were performed using the GATK HaplotypeCaller, according to previous studies^7,15^. SV calling was performed using the DELLY program, with default parameters^16^. TE insertion calling was performed using PopoolationTE2, with default parameters^17^.

### RNA-seq analysis

RNA-seq data analysis was performed using the HISAT, StringTie and Ballgown^18^. Cleaned reads were mapped against the peach reference genome using HISAT2 (Version 2.0.5), with default parameters^19^. Transcript assembly and the estimation of the transcript abundances were performed using the Stringtie program^20^. DEG analysis was carried out using the R package ballgown^21^.

### GO and KEGG analysis

The coding sequences of DEGs were extracted from the reference genome to be used as the input for the GO and KEGG analyses. GO and KEGG analyses were performed using the gene-list enrichment module in the KOBAS 3.0 program, based on a Fisher’s exact test^22^.

## Results

### Impacts of hairless bud mutation on agronomic traits

To identify the impacts of the hairless bud mutation on fruit quality, the sugar content and fruit size were measured. We found that the average fruit weight of the hairless bud mutation was 173.8 ± 13.5 g, which was significantly lower than the wild types (228.8 ± 12.4 g) (Fig. 1a and b), suggesting that the bud mutation may have negative impacts on the fruit size. In contrast, the soluble solid content was upregulated in the hairless bud mutation (14.8% in the hairless bud mutation versus 12.8% in the wild type) (Fig. 1c), implying that the increase in the sugar content was caused by the hairless bud mutation. Using the HPLC method, we identified four major sugars in peach fruit flesh, including sucrose, glucose, fructose, and sorbitol^23^which were the major contributors for SSC. Compared with the wild type, glucose and fructose were upregulated, while sucrose and sorbitol were downregulated in mutation (Fig. 1d). Collectively, our results suggest that the somatic mutation has a complex impact on several agronomic traits, not only hairless, implying that the genes participate in the pathways involved in several traits have been impacted.

### Transcriptome and genome sequencing data

To understand the impacts of the hairless mutation on gene expression, RNA-seq analyses were performed using the wild type (‘Zhong Tao 5’) and mutation (bud mutation of ‘Zhong Tao 5’) fruits, with three replicates. In total, six sequence libraries were sequenced. Finally, 24.6 Gb pair-end sequences with an average Q30 of 96.0% were generated using the Illumina platform (Table 1). After removing low-quality data and aligning against the reference genome, the average mapping rate was 93.8%, indicating that the dataset met the criteria for further analyses. Remarkably, the mapping rates of the wild type and mutation samples harbored no significant differences (94.3% versus 93.3%), suggesting that the bud mutation for hairless fruit may have a slight impact on coding sequences.

To verify the impacts of the bud mutation on the peach genome, high-depth genome sequencing was also conducted for the wild type and bud mutation samples. Finally, 10.8 Gb and 11.2 Gb pair-end sequences were generated for the wild type and hairless bud mutation (Table 1), respectively. After aligning against the reference genome, final mapping ratios of 95.4% and 94.0%, depths of 40.5 and 40.1, and genome coverages of 97.3% and 97.2% for the wild type and bud mutation were revealed (Table 1), supporting the slight difference at the genome level.

**Table 1:** Summary of sequencing data.

	Sample	Total reads	Total mapped	Multiple mapped	Uniquely mapped
RNA-seq	Mutation_1	25,866,622	24,327,901 (94.05%)	666,008 (2.57%)	23,661,893 (91.48%)
	Mutation_2	25,668,532	24,149,455 (94.08%)	654,054 (2.55%)	23,495,401 (91.53%)
	Mutation_3	27,638,476	26,193,040 (94.77%)	691,265 (2.5%)	25,501,775 (92.27%)
	WT_1	26,163,454	23,597,044 (90.19%)	606,531 (2.32%)	22,990,513 (87.87%)
	WT_2	26,426,008	24,841,937 (94.01%)	673,254 (2.55%)	24,168,683 (91.46%)
	WT_3	28,695,188	27,507,060 (95.86%)	626,301 (2.18%)	26,880,759 (93.68%)
WGS	Sample	Total reads	Map ratio (%)	Depth (×)	Coverage (%)
	Mutation	71,516,178	94.07	40.13	97.96
	WT	71,530,222	95.41	40.51	97.96

### Genomic changes in bud mutation

**Fig. 2:**
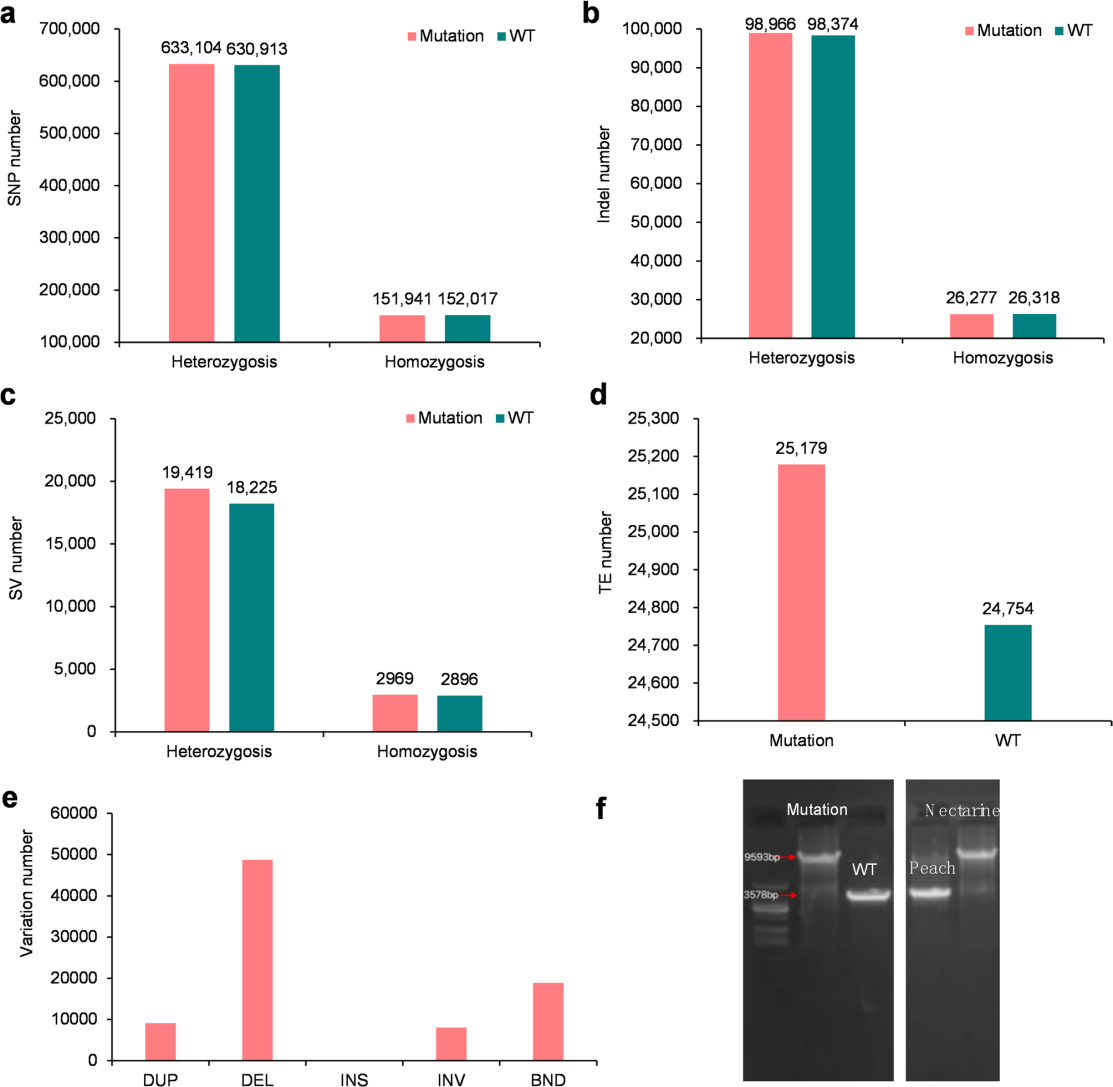
The summary of genomic variations in wild type and bud mutation. The number of heterozygosis and homozygosis SNP (**a**), indel (**b**), SV (**c**), and TE (**d**) in wild and bud mutation. (**e**) The number of DUP, DEL, INS, INV, and BND variations in bud mutation. (**f**) The PCR products for the *PpMYB25* in mutation and WT accessions. The TE insertion was identified.

To reveal the genomic changes in bud mutations, we constructed an integrative variation map based on deep sequencing data, including the genome-wide single nucleotide polymorphism (SNP), insertion and deletions (indels), structural variations (SVs), and transposable elements (TEs). Finally, a total of 825,750 SNPs, 129,688 indels, 25,327 SVs, 2,122 CNVs, and 48,806 TEs were identified (Fig. 2). A total of 823,635 (99.7%) SNPs, 129,137 (99.6%) indels, 24,060 (95.0%) SVs, 48,381 (99.1%) TEs were shared by mutation and wild type, suggesting most of genomic segments were not changed in mutation accession. We found that the mutation accession harbored 2,115 more SNPs than the wild type (785,045 SNPs in mutation versus 782,930 in wild type) (Fig. 2a; Supplementary Table S1). Moreover, most of the 2,115 SNPs were heterozygous, suggesting that the hairless mutation contained a high number of variations. Regarding indels, the mutation harbored 551 more than the wild type (125,243 indels in mutation versus 124,692 indels in wild type) (Fig. 2b). Most of the indels in the mutation but absent in the wild type were heterozygous (Fig. 2b). Similarly, we found that the mutation contained more SVs than the wild type (22,388 SVs in mutation versus 21,121 SVs in wild type) (Fig. 2c). Among the SVs, we found that deletion (DEL) and translocation (BND) were dominant, followed by duplication (DUP) and inversion (INV), successively (Fig. 2e). Remarkably, insertion (INS) was absent in the mutation accession (Fig. 2e). This result was different to that found in the natural population, in which the INS number was higher than the DUP and INV number, suggesting that the hairless somatic variation bias leads to the loss of sequences, rather than the addition of new sequences. Regarding TEs, we identified more TE insertions in the mutation (25,179) than in the wild-type accession (24,754) (Fig. 2d).

Notably, previous studies have found that a 5836 bp Ty1-copia retrotransposon insertion in *PpMYB25* results in the hairless phenotypes^24]– [25^. In this study, we also found this TE in the mutation accession (Fig. 2f and Supplementary Figs. 1 and 2), suggesting the key variation underlying the hairless bud mutation and further suggesting the function of *PpMYB25* in the formation of nectarine. Collectively, our results indicated the extensive variation across the genome for bud mutations other than point mutations, and that a TE insertion in *PpMYB25* leads to the hairless phenotype in this mutation. Moreover, the majority of genomic variation was biased towards heterozygosis, which might explain the extensive genomic variations but fewer phenotype changes for the somatic mutation.

### Differential expression genes between wild type and bud mutation

**Fig. 3:**
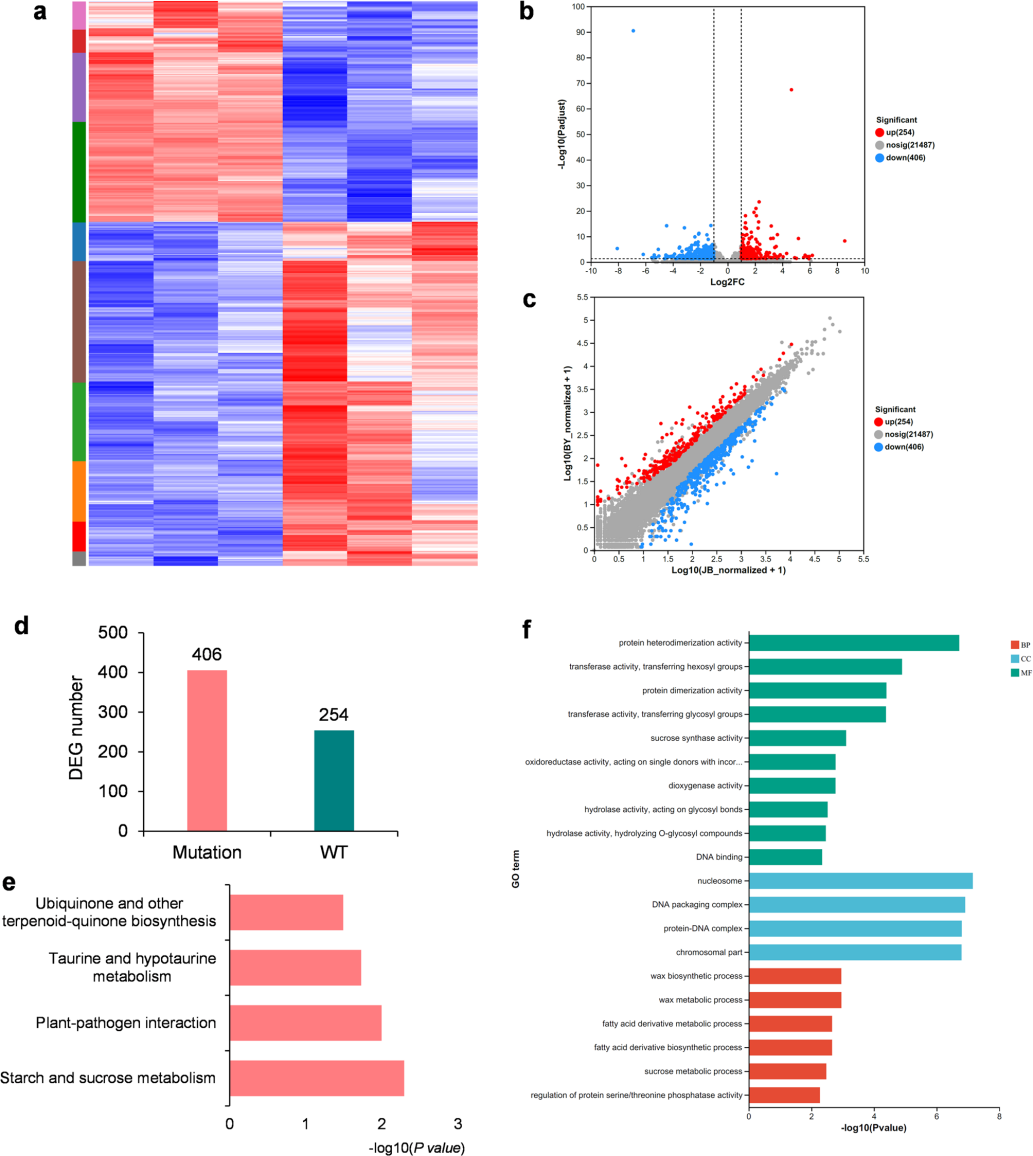
Summaries of differentially expressed genes (DEGs) and function enrichment. (**a**) Heatmap of all expressed genes. (**b**) Volcano map of all genes and DEGs. (**c**) Fold changes of all genes and DEGs. (**d**) Comparison of DEGs in the wild type and bud mutation. (**e**) KEGG enrichment of DEGs. (**f**) GO enrichment of DEGs.

A total of 22,151 expressed reference genes were identified in the wild type and bud mutations. Of these, 21,480 expressed in wild type and 21,354 expressed in bud mutation, resulting in 20,683 expressed in both of them (Fig. 3a and b). Of which, 583 genes involved in phenylpropanoid biosynthesis, biosynthesis of secondary metabolites, flavonoid biosynthesis, stilbenoid, diarylheptanoid and gingerol biosynthesis were specific expressed in mutation (Supplementary Table S2), suggesting the possible pathway influenced by the bud mutations. In addition, by comparing the average expression level of the genome-wide genes, we found that the expression level at the genome level was similar between the wild type and bud mutation (Fig. 3c), suggesting that the gene expression was not as affected by the bud mutation. Remarkably, we also identified 1005 novel genes that were not present in the reference genome; of these, 917 were expressed in the wild type, 924 were expressed in the bud mutation, and 836 were expressed in both.

Compared with the wild type, a total of 660 differentially expressed genes (DEGs) with |log2FC| > 1 and adjust *p* < 0.05 were identified in the bud mutation samples, including 634 references genes and 26 novel genes (Fig. 3b and c). Among DEGs in the mutation sample, 254 were upregulated and 406 were downregulated (Fig. 3d; Supplementary Table S3), suggesting that suggesting that the bud mutation generate more negative regulations on gene expression in peach.

To determine which biological processes were impacted by the bud mutation, Gene Ontology (GO) and Kyoto Encyclopedia of Genes and Genome (KEGG) analyses for DEGs were performed^26^. Based on Fisher’s exact test, a total of four KEGG pathways were overrepresented (*p* < 0.05) (Fig. 3e), including the ‘starch and sucrose metabolism’ pathway, ‘plant-pathogen interaction’ pathway, ‘taurine and hypotaurine metabolism’ pathway, and ‘ubiquinone and other terpenoid quinone biosynthesis’ pathway. A total of 78 GO terms involved in plant development, response to stress, metabolite synthesis, etc, were identified (Fig. 3f; Supplementary Table S4). These related biological processes might be impacted by the hairless bud mutation directly or indirectly.

### Source-synthesis-related genes

Remarkably, we found that the most significantly enriched KEGG pathway was the ‘starch and sucrose metabolism’ pathway (map00500) (*p* = 0.005), involving nine genes; of these, five were upregulated and four were downregulated. The content and balance of sugar and acid were important for fruit flavor, and the latter included sucrose, glucose, fructose, and sorbitol. Previous studies have stated that the major sugar conferring the sweet taste is sucrose, which is increased significantly during fruit ripening. Here, we found that the genes related to sucrose metabolism presented a significant difference in expression between the wild type and bud mutation. Notably, we found that two genes, *PpSUS3* and *PpSUS4*, encoding a putative sucrose synthase were involved in the catalyzing of the reversible reaction between sucrose and glucose^27^and that fructose was significantly upregulated in the hairless mutation (*p* < 0.05). Consistently, declines in the sucrose content was observed in the hairless mutation (Fig. 1d). Moreover, based on our previous RNA-seq data, we found that the expressions of *PpSUS3* and *PpSUS4* were downregulated during fruit ripening^7^suggesting that *PpSUS3* plays a putatively negative role in the regulation of total sugar accumulation in peach.

**Fig. 4:**
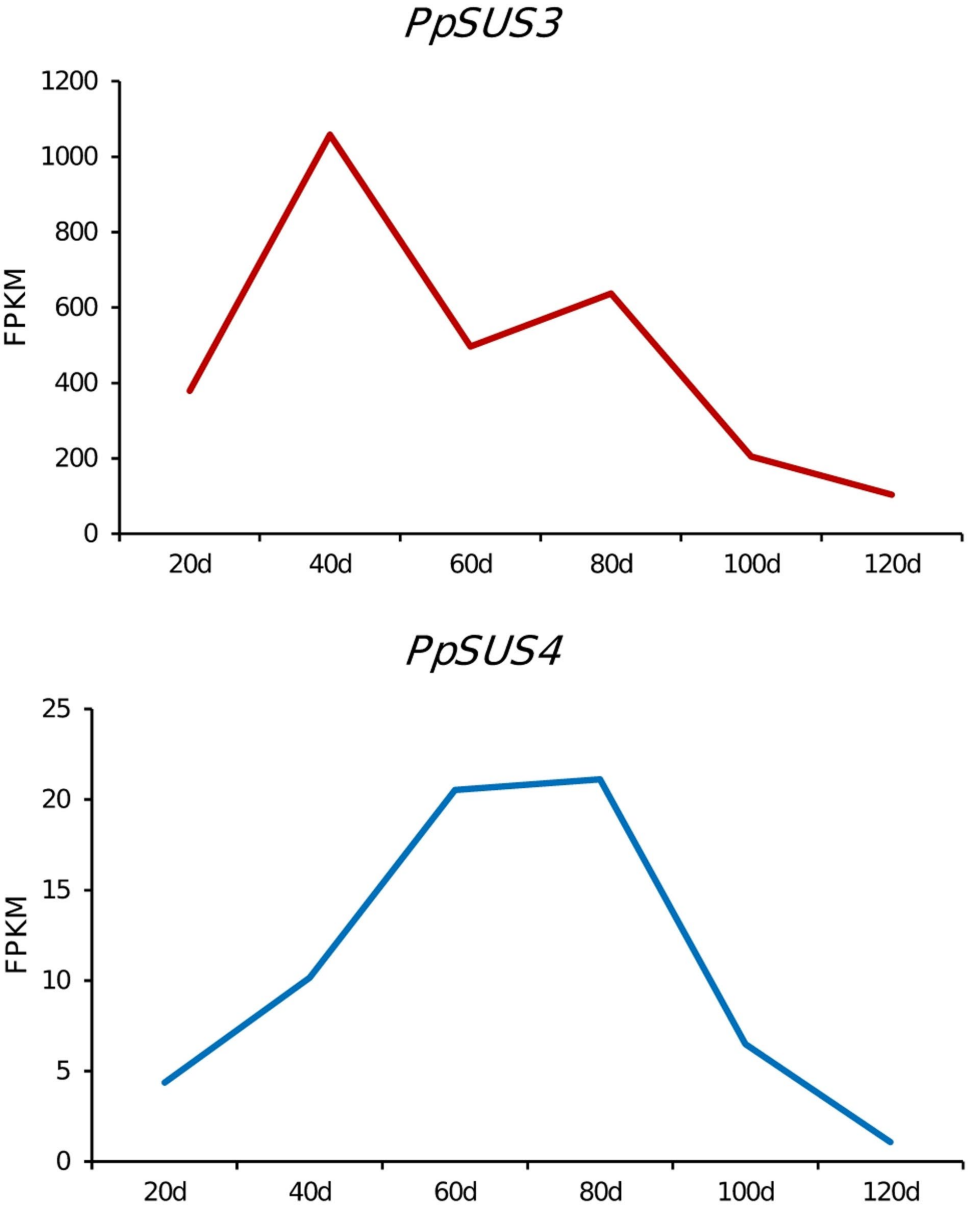
The expression of *PpSUS3* and *PpSUS4* during peach fruit development.

**Fig. 5:**
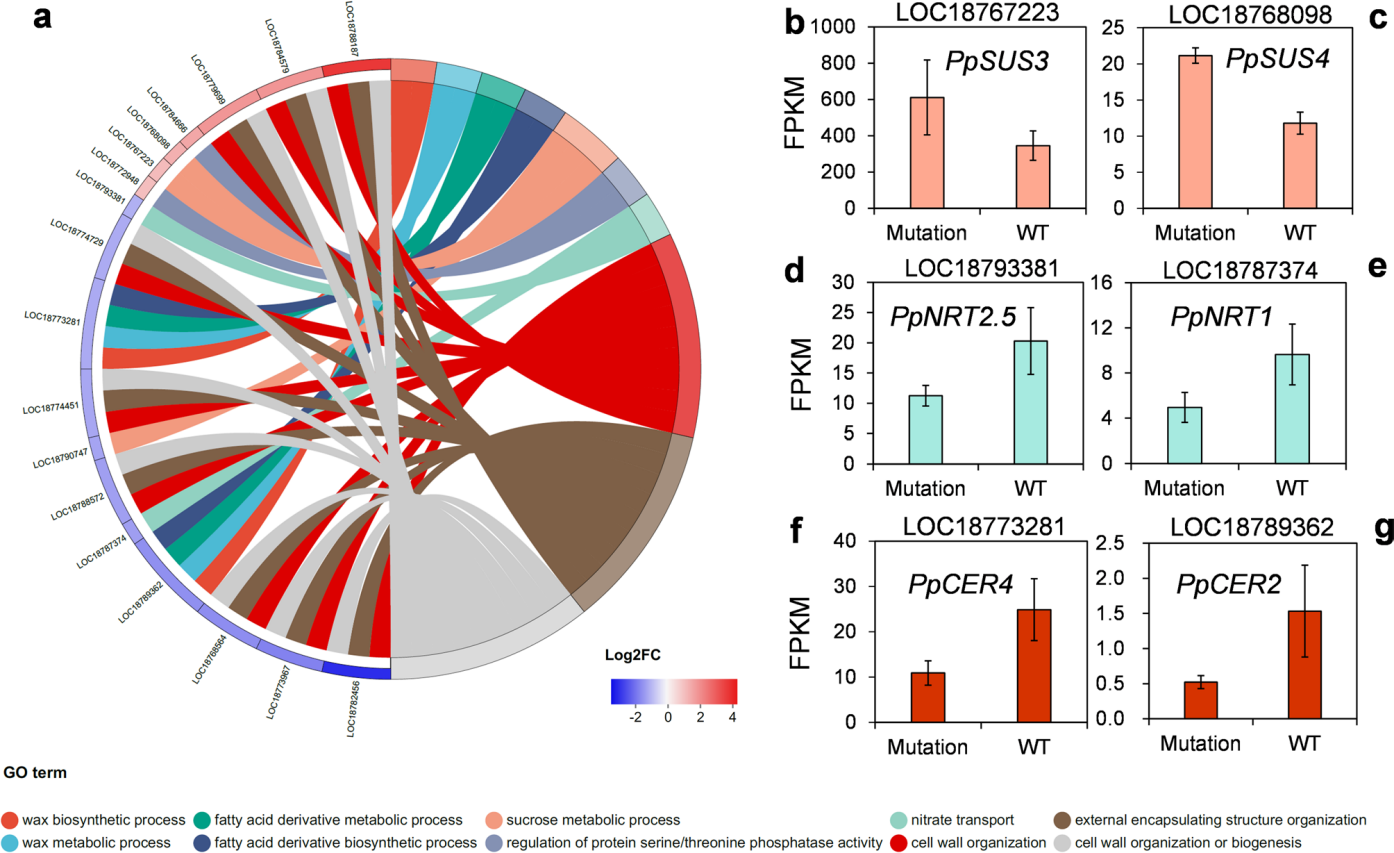
Key genes and biological process related to the phenotype changes. (a) Circus plot for GO terms. (b-g) Expressions of key genes in the wild type and bud mutation.

Similarly, in GO terms, we also found that the ‘sucrose metabolic process’ (GO:0005985) was significantly overrepresented (*p* < 0.05) (Fig. 5a), as well as two sucrose synthase genes, namely *PpSUS3* and *PpSUS4*. These two genes are also involved in the KEGG pathway, and in starch and sucrose metabolism. Both of them were highly expressed in the mutation accession compared with the wild type (Fig. 5b-5c). The sucrose synthase genes were found to play important roles in the regulation of the sugar content in plants, such as tomato and sweet oranges. Previous study has stated the *SUS* genes catalyze the reversible reactions between sucrose and fructose^28^. In this study, the upregulation of *PpSUS3* and *PpSUS4* was found to potentially underlie the decrease of sucrose and increase of fructose and then the higher SSC in the mutation accession. In addition, a total of 292 differentially expressed transcription factors were identified in mutant accession (Supplementary Table S5). Of which, 36 genes enriched in plant hormone signal transduction and SNARE interactions in vesicular transport pathway, suggesting the pathways influenced by bud mutation at transcriptional level.

### Nitrate-transport-related genes

Based on the phenotype observation, we found that the fruit size of the hairless mutation was significantly smaller than the wild type (Fig. 1b). As an essential nutrient for plants, nitrogen is important for the formation of plant organs, including the fruits. Via the functional annotation analyses of DEGs, we found that two genes involved in nitrate transport, namely *PpNRT2.5* and *PpNRT1*, were significantly downregulated in the mutation accession (Fig. 5d and e). NRT genes encode the nitrate transporter proteins, which participate in the uptake and distribution of nitrogen^29^. The lower expression of *PpNRT2.5* and *PpNRT1* might have led to the decline in the nitrogen absorptive capacity and subsequently generated the negative impacts had on the fruit size.

### Wax-biosynthesis-related gene

The hairless mutation presented two major characteristics that were not exhibited by the wild type: hairlessness and a waxy skin. Although our study was focused on the impacts of somatic variation on fruit flesh, we also identified wax-biosynthesis-related genes in DEGs. According to the gene enrichment analyses, the wax biosynthesis and metabolic pathways were overrepresented (*p* < 0.05) (Fig. 5a). Of these, two important genes, namely *PpCER3* and *PpCER4*, were revealed to have a higher expression level in the wild type than in the mutation accession (Fig. 5f and g). *CER3* and *CER4* encoded the alcohol forming fatty acyl-CoA reductase, which is involved in cuticular wax biosynthesis^30^.

## Discussion

Somatic mutation is an important resource for generating new variations, and is an essential breeding solution for several plant species, such as sweet orange and jujube^3]– [4^. Hairless bud mutations were found to be the high-frequency somatic mutations for peach. A previous study found that nectarine (hairless) harbors a higher level of SSC content and a smaller fruit size^31^making the hairless mutation an excellent breeding material when aiming to achieve a high sugar content. In this study, we found that the hairless bud mutation could generate a higher sugar content and a smaller fruit size, supporting the findings in nectarine.

In this study, we found that the bud mutation could lead to the genome-wide extensive sequence and expression changes in peach. In previous study, by analyses genome sequencing data of 74 somatic mutation accessions from ‘Fuji’ apple, a total of 68,965 somatic SNPs, 27,757 somatic indels, and 1848 somatic SVs were identified^32^supporting the extensive genomic impacts by bud mutation. Notably, only 532 deleterious SNP were identified in 74 mutation accessions, suggesting the deleterious somatic variants were rare in apple. In sweet orange, only 8,628 somatic SNP, 2,818 somatic InDels, and 2,321 somatic SVs were identified from 104 somatic mutation accessions^3^. The number of somatic variations was significantly less than apple and peach in study, indicating the variation of somatic mutation was different in different species.

We found an LTR insertion in the third exon of *PpMYB25* underlying the hairless phenotype in this bud mutation, consistent with previous studies using population mapping^24]– [25^. We found that the two sucrose synthase genes, namely *PpSUS3* and *PpSUS4*, were upregulated by the hairless bud mutation. Sucrose synthase is a key enzyme involved in the metabolism of sucrose, and catalyzes the translations from sucrose to fructose. The upregulation of *PpSUS3* and *PpSUS4* in the bud mutation contributed to the catabolism of sucrose. This result explained the decrease in sucrose and increase in fructose in the hair-less bud mutation, providing new insights into the higher SSC content in nectarine than peach. In addition, we also found that two nitrate transporter genes were downregulated by the hairless bud mutation. The low expression level of nitrate transporter in the hairless bud mutation might have led to the lower level of nitrogen and subsequent small fruit size. Collectively, our results reveal that the hairless bud mutation does not constitute a point change, but instead wide changes across the genome. The hairless bud mutation could have extensive impacts on sequence polymorphism and gene expression.

## Electronic supplementary material

Below is the link to the electronic supplementary material.

Supplementary Material 1

Supplementary Material 2

## Data Availability

The RNA and DNA sequencing data have been deposited in the NCBI Sequence Read Archive (SRA) under accession PRJNA1028150. The peach reference genome (v2.0) used in this study is available from Genome Database for Rosaceae (https://www.rosaceae.org/species/prunus_persica/genome_v2.0.a1). Gene expression data in Figure 4 were derived from our previous data under accession PRJNA694195.
