# Supplementary material for: Genomic and transcriptomic analyses provide new insights into the complex impacts of bud mutation in peach
Source: Sci Rep. 2025 Jul 2;15:22951. doi: 10.1038/s41598-025-07225-w (PMC12216846; doi:10.1038/s41598-025-07225-w)
Supplement: Supplementary file 2 — Supplementary Material 2 [file 41598_2025_7225_MOESM2_ESM.docx]

**Supplementary Figure S1** The PCR gel plot for the genotyping of *PpMYB25*. M, marker; Mut, mutation; WT, wild type; P, peach; N, nectarine. P and N were used as the positive and negative controls. To make the figure readable, the edge at left of gel and a blank lane at right of gel has been cropped.

*PpMYB25*

Bud mutation


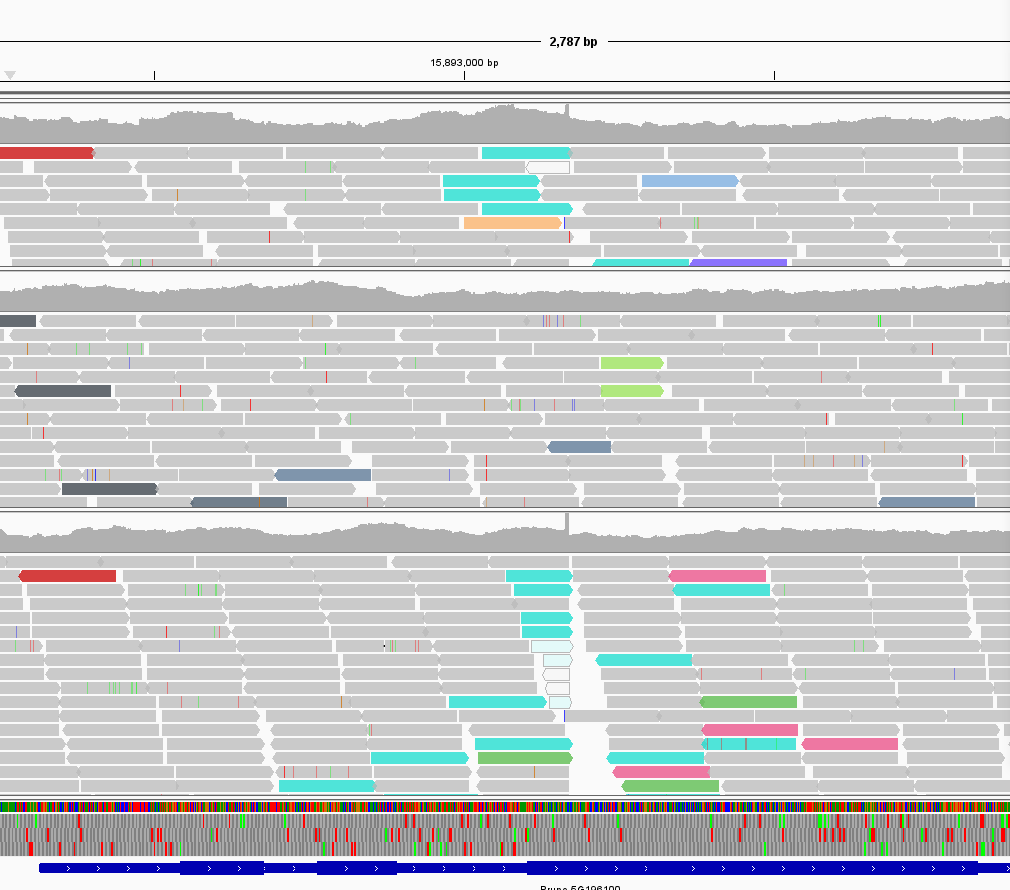


Diao Zhi Bai

Peach

Sunfre

Nectarine

**Supplementary Figure S2** The alignment of reads around *PpMYB25* in hairless bud mutation, peach and nectarine. Red arrows indicate the position of TE insertion.
